# Cytokeratin-14 contributes to collective invasion of salivary adenoid cystic carcinoma

**DOI:** 10.1371/journal.pone.0171341

**Published:** 2017-02-02

**Authors:** Xiao-lei Gao, Jia-shun Wu, Min-xin Cao, Shi-yu Gao, Xiao Cen, Ya-ping Jiang, Sha-sha Wang, Ya-jie Tang, Qian-ming Chen, Xin-hua Liang, Yaling Tang

**Affiliations:** 1 State Key Laboratory of Oral Diseases West China Hospital of Stomatology, Sichuan University, Chengdu, People’s Republic of China; 2 Key Laboratory of Fermentation Engineering, Ministry of Education, Hubei University of Technology, Wuhan, People’s Republic of China; 3 Department of Oral and Maxillofacial Surgery, West China Hospital of Stomatology, Sichuan University, Chengdu, People’s Republic of China; 4 Department of Oral Pathology, West China Hospital of Stomatology, Sichuan University, Chengdu, People’s Republic of China; NIDCR/NIH, UNITED STATES

## Abstract

Collective invasion of cells plays a fundamental role in tissue growth, wound healing, immune response and cancer metastasis. This paper aimed to investigate cytokeratin-14 (CK14) expression and analyze its association with collective invasion in the invasive front of salivary adenoid cystic carcinoma (SACC) to uncover the role of collective invasion in SACC. Here, in the clinical data of 121 patients with SACC, the positive expression of CK14 was observed in 35/121(28.93%) of the invasive front of SACC. CK14 expression in the invasive front, local regional recurrence and distant metastasis were independent and significant prognostic factors in SACC patients. Then, we found that in an ex vivo 3D culture assay, CK14 siRNA receded the collective invasion, and in 2D monolayer culture, CK14 overexpression induced a collective SACC cell migration. These data indicated that the presence of characterized CK14+ cells in the invasive front of SACC promoted collective cell invasion of SACC and may be a biomarker of SACC with a worse prognosis.

## Introduction

Salivary adenoid cystic carcinoma (SACC) represented approximately 10% of all salivary neoplasm. SACC is by insidious invasion into adjacent tissue and hematogenous spread to distant organs [1–2]. Local invasion and distant metastasis are the main reasons to lead to the low overall survival, however, the molecular mechanism of invasion and metastasis in SACC still need to be investigated.

It is commonly conceptualized that carcinoma progression begins with single cell invasion, in which cancer cell disrupts tight intercellular junction and gains a mesenchymal phenotype by epithelial-mesenchymal transition (EMT) [3–5]. However, histomorphologically, one of the frequently observed invasion models is collective invasion that retains cell-cell adhesions, migrates in the same direction at a similar speed and affects one another while migrating. This coordinated movement of multicellular unit has three major forms of multicellular clusters, strands, or tubes, and is considered as a fundamental process during cancer invasion [6–9]. Recent studies have uncovered that collective cell invasion depends on not only cell-intrinsic mechanism by interactions between the leader cells and the follower cells, but extracellular mechanism by bi-directional interplays between tumor cell and the microenvironment, especially, proteolysis-mediated matrix remodeling and contractility-mediated matrix remodeling[10–11]. While tumor cell collective invasion has been experimentally modeled, clinically-relevant drivers of the process are only now beginning to be identified.

CK14, one of about 20 different cytokeratin isotypes of human cells, is the intermediate filament protein characteristic of epithelial cells. In various kinds of human tumors, the appearance and increasing expression of CK14 were strikingly associated with higher grade and stage of carcinoma, with varying degrees of unfavourable prognosis[12–13].In lung squamous cell carcinoma(LSCC), CK 14 was expressed in the tumor cell nests showing stromal invasion with fibrosis and lymph node metastases, indicating that CK14 involved in proliferation and metastasis of LSCC [14]. Importantly, CK14^+^ cells were the leader cells of collective invasion of breast cancer, which lead the migration of CK14^−^ cells and were migratory, protrusive, and cell–cell cohesive [15]. These findings made us ask whether CK14 plays an important role in collective invasion of SACC.

Here, we found that the positive expression of CK14 in the invasive front was significantly associated with local regional recurrence, distant metastasis and poor prognosis of SACC patients. In an ex vivo 3D culture assay CK14 siRNA receded the collective invasion, and in 2D monolayer culture, CK14 overexpression induced a collective SACC cell migration, suggesting that CK14 promoted the collective invasion of SACC.

## Material and methods

### Ethics statement

The protocol of the study was approved by the Institutional Ethics Committee of the West China Medical Center, Sichuan University, China.

### Patients’ enrollment

One hundred and twenty-one cases of SACC without treatment before surgery were enrolled at Department of Oral and Maxillofacial Surgery, West China Hospital of Stomatology, Sichuan University between 1996 and 2005. Demographic and other variables were shown in Table 1. In addition, 10 of normal salivary gland were included in this study. All the data were obtained with the person’s understanding that it might be published. The written informed consents of participants were obtained in this study through the signatures of participants. All was in accordance with the rules of the Institutional Ethics Committee of the West China Medical Center, Sichuan University, China.

**Table 1:** Clinicopathologic features of the SACC patients and their primary tumors and their association with CK14 expression (*n* = 121).

Clinicopathological features	No. of cases	K 14 expression		*P-value*
		Negative(*n* = 86)	Positive(*n* = 35)	
Age(years) at diagnosis	121			1.0000
<50	45	32	13	
> = 50	76	54	22	
Sex	121			0.1063
Female	67	52	15	
Male	54	34	20	
Complaints, months	121			0.0753
<12	64	50	14	
≥12	57	36	21	
Site	121			0.0088
Minor salivary gland	63	38	25	
Major salivary gland	58	48	10	
Histological subtype	121			0.0173
Tubular/ Cribiform	85	66	19	
Solid	36	20	16	
TNM stage	121			0.0762
Ⅰ-Ⅱ	34	20	14	
Ⅲ-Ⅳ	87	66	21	
Perineural invasion	100			0.0441
Yes	47	29	18	
No	53	43	10	
Local regional recurrence	121			0.0052
Yes	30	15	15	
No	91	71	20	
Distant metastasis	103			0.0410
Yes	39	9	12	
No	64	44	20	

Pathologic slides were assessed by one pathologist (Tang YL) who was not given any information on the previous diagnosis of patients. The World Organization’s International Histological Classification of Salivary Gland Tumors was applied to determine the histological classification of each tumor [1]. The UICC TNM classification of malignant tumor was used to detect tumor clinic stage [2].

The area of the invasive front denotes as “tumor buds”, where groups of cancer cells are signaled to move collectively from their original site and invade a surrounding matrix environment[16–17]. We chose the area of the invasive front from each primary tissue, which was employed for immunohistochemical analysis.

### Immunohistochemistry (IHC)

Immunohistochemical detection of CK14 was carried on using an anti-CK14 antibody (ID 10143-1-AP, Proteintech, USA, 1:200) as the previous publication [18]. The stain intensity was graded from negative, weak positive, and strong positive, compared with background staining. The ratio of CK14-positive cells was expressed as the percentage of 1000 tumor cells counted within 4~6 microscopic fields at ×200 magnification and semi-quantitatively graded as follows: negative (0% to 9%), and positive (>10%). Quantitation of positive cells was done by 2 independent observers (Wang SS and Gao SY), who had been trained for 2 weeks and had a unified diagnosis criterion. Once they had divergence and the oral pathologist (Tang YL) would give final diagnosis.

### Culture of tumor organoids

Fresh tissues of 5 patients with salivary adenoid cystic carcinomas were obtained after giving informed consent. The protocol of the study was approved by the Institutional Ethics Committee of the West China Medical Center, Sichuan University, China. 5 patients (3 male and 2 female; median age, 52 years; range 48–62) without preoperative chemotherapy, hormone therapy or radiotherapy were recruited. Among 5 cases, 2 cases in parotid gland was cribriform, 1 in palate was tubular, and 1 in submaxillary gland and 1 in cheek was solid. 5 patients had no perineural invasion, local regional recurrence and distant metastasis.

Briefly, the diagnosis of each tumor sample was re-reviewed before receipt with limited prespecified clinical information provided with each sample. The invasive front of the tumor was detected by frozen section, and an immediately adjacent piece (3–20 mm^3^) was obtained for culture. The tumor sample was kept in cold Dulbecco’s modified Eagle’s medium (DMEM) of an ice box during transit. The sample was rinsed with antibiotic wash, minced, and then digested in collagenase with or without trypsin. Tumor organoids were treated with DNase and separated out by differential centrifugation at 100 g×5 min. Tumor organoids were then allocated to Matrigel to culture. Medium was replaced every 3 to 4 days

### Inhibition of CK14 expression by RNAi

The cells were transfected with 100 nM of CK14 siRNA in serum-free Opti-MEM medium with the use of Lipofectamine 2000 (Invitrogen, Carlsbad, CA, USA) as suggested by the manufacturer’s instructions. The target sequence was: siRNA1 sense, GAGUUGASACCUGCGCAUGAtt; antisense, UCAUGCGCAGGUUCAACUCtg; siRNA2sense, GCGATTAATCCCTGCATA AGAAGGAGACA,antisense,CGCTATACCGGTAAGGCTGAGTGAAGAGAAGG.

### Statistical analysis

All the statistical analyses were performed using SPSS 13.0 for Windows (SPSS Inc., Chicago, IL, USA). The association betweenCK14 expression and clinicopathological factors was analyzed by the Chi-square test. The Kaplan–Meier method was applied to calculate overall survival rates. Differences between the groups were evaluated using the log-rank test. The prognostic factors were examined by univariate and multivariate analyses of Cox’s proportional hazards model. A value of *p* < 0.05 was considered statistically significant.

## Results

### CK14 expression in invasive front associated with the invasion and metastasis, and poor prognosis of SACC patients

**Fig 1:**
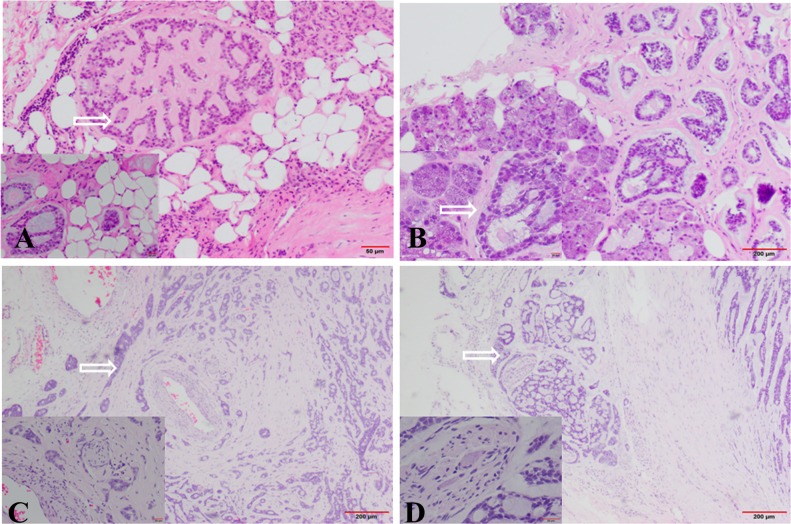
SACC collective cells invaded into normal tissues under light microscopy with H&E staining. (A) Arrow showed invasion into fat tissue (HE×200; ×400). (B) Arrow showed invasion into gland (HE×200; ×400). (C) Arrow showed invasion into blood vessel (HE×200; ×400). (D) Arrow showed invasion into nerve tissue (HE×200; ×400). The invasive cells were aggregated or clustered, and not single cells.

In SACC samples, the outer edges of tumor were no envelope and highly irregular when observed at low power of light microscope. Associated with the edges was plenty of cohesive micronodules or buds that surrounded the primary tumor mass. And we often observed these micronodules invasion to muscle, blood vessel, nerve, and normal salivary gland (Fig 1). These areas were usually regarded as the invasive fronts. Compared with tumor center, Ki67 staining showed an increase in proliferation at the invasive fronts.

**Fig 2:**
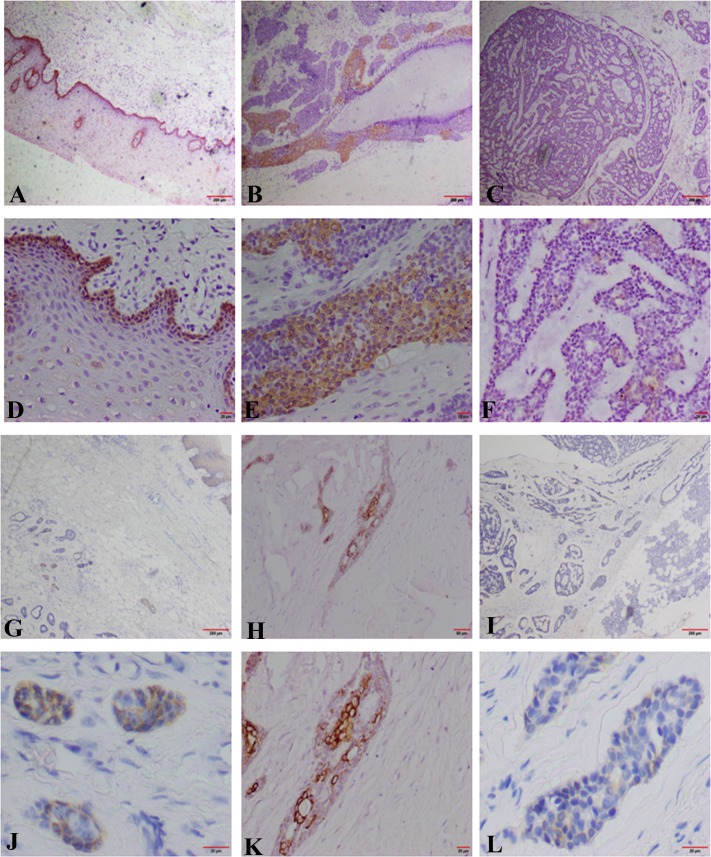
CK14 photomicrographs in the invasive front of SACC with IHC. (A, D) Positive expression of CK14 in oral mucuous tissue (A×100; D×400); (B, E) Positive expression of CK14 in the invasive front of SACC with solid pattern (B×100; E×400); (C, F) Positive expression of CK14 in the invasive front of SACC with cribriform pattern (C×100; F×400). The CK14 positive expression in invasive front in cribriform pattern was lower than that in solid pattern of SACC; (G, J) Positive expression of CK14 in the invasive front of SACC invasion to oral mucuous tissue (G×100; J×400); (H, K) Positive expression of CK14 in the invasive front of SACC invasion to blood vessel(H×200; K×400); (I, L) Positive expression of CK14 in the invasive front of SACC invasion to gland (I×100; L×400).

To further investigate the relationship between collective invasion and clinic-pathological factors of patients, we applied the invasive fronts to carry out immunohistochemistry staining of CK14, the marker of collective movement, in 121 specimens of salivary SACC, and 10 of normal salivary gland were used as the control. The result demonstrated that the positive staining of CK14 reactivity was usually located in the cytoplasm of cancer cells (Fig 2). There was CK14 positive expression in 35/121(28.93%) specimens of salivary SACC and 10/10 (100%) expression in the normal salivary gland tissue. The correlation between the CK14 positive expression in invasive front and clinic-pathologic factors of SACC was further analyzed (Table 1). The data showed that the CK14 positive expression in invasive front of minor salivary gland was higher than in major salivary gland (*P* = 0.0088). The CK14 positive expression in invasive front in 22.35% (19/85) of cases with tubular or cribriform pattern was much lower than that in solid pattern 44.44% (16/36) of SACC (*P* = 0.0173, Fig 2B and 2C). Importantly, CK14 positive expression of invasive front was significantly associated with perineural invasion, local regional recurrence and distant metastasis (*P* = 0.0441, *P* = 0.0052, *P* = 0.0410, respectively). However, there was no significant association of CK14 positive expression of invasive front with age, sex, TNM stage and complaints of patients.

Survival curves were computed with the Kaplan-Meier method and compared between groups by using the log-rank test. The cases with CK14 positive expression in invasive front had a poorer prognosis than those with CK14 negative expression (*P*<0.05). The univariate and multivariate analyses of factors related to patient prognosis were carried on in all patients. The univariate analysis showed that site, histological subtype, TNM stage, perineural invasion, local regional recurrence, distant metastasis and CK14 expression in invasive front were significantly associated with patient survival (*P*<0.05, Table 2). All the variables with *P*<0.05 through the univariate analysis were applied for multivariate analysis. The Cox’s proportional hazards model confirmed that CK14 expression in invasive front, local regional recurrence and distant metastasis were three independent prognostic factors in SACC patients (Table 3).

**Table 2:** Univariable survival analysis of clinical and pathological data of 121 adenoid cystic carcinoma of salivary gland.

Clinicopathological features	No. of cases	5 years Survival, %	*p*-value	10 years Survival,%	*p*-value
Age(years) at diagnosis	121		0.135		0.083
<50	45	65.1		33.8	
> = 50	76	48.6		27.9	
Sex	121		0.076		0.092
Female	94	64.8		34.9	
Male	54	47.9		28.4	
Complaints, months	121		0.756		0.115
<12	64	48.6		33.2	
≥12	57	58.6		34.2	
Site	121		0.007		0.003
Minor salivary gland	63	44.2		16.8	
Major salivary gland	58	59.8		34.1	
Histological subtype	121		0.001		0.009
Tubular/ Cribiform	85	59.7		39.4	
Solid	36	24.6		0.0	
Resection margins	121		0.095		0.079
Free	88	57.9		30.8	
Affected	33	41.8		21.3	
TNM stage	121		0.008		0.003
Ⅰ-Ⅱ	34	65.4		39.4	
Ⅲ-Ⅳ	87	44.8		23.8	
Perineural invasion	100		<0.001		<0.001
Yes	53	27.3		19.5	
No	47	36.1		30.5	
Local regional recurrence	121		<0.001		<0.001
Yes	30	32.7		21.6	
No	91	61.2		31.5	
Distant metastasis	103		<0.001		<0.001
Yes	39	12.8		0.0	
No	64	58.2		31.7	
CK14 expression	121		<0.001		<0.001
Positive	35	22.6		8.9	
Negative	86	77.4		41.7	

**Table 3:** Independent significant prognostic factors after cox multivariate survival analysis of adenoid cystic carcinomas of salivary gland.

Variable	Categories	Relative risk (95% confidence interval)	*p*-Value
regional recurrence	present	2.3 (1.3–4.1)	<0.001
Distant metastasis	with	3.6 (2.1–6.1)	<0.001
CK14 expression	positive	2.2(1.1–2.9)	<0.001

### CK14 siRNA receded the collective invasion in an ex vivo 3D culture assay

**Fig 3:**
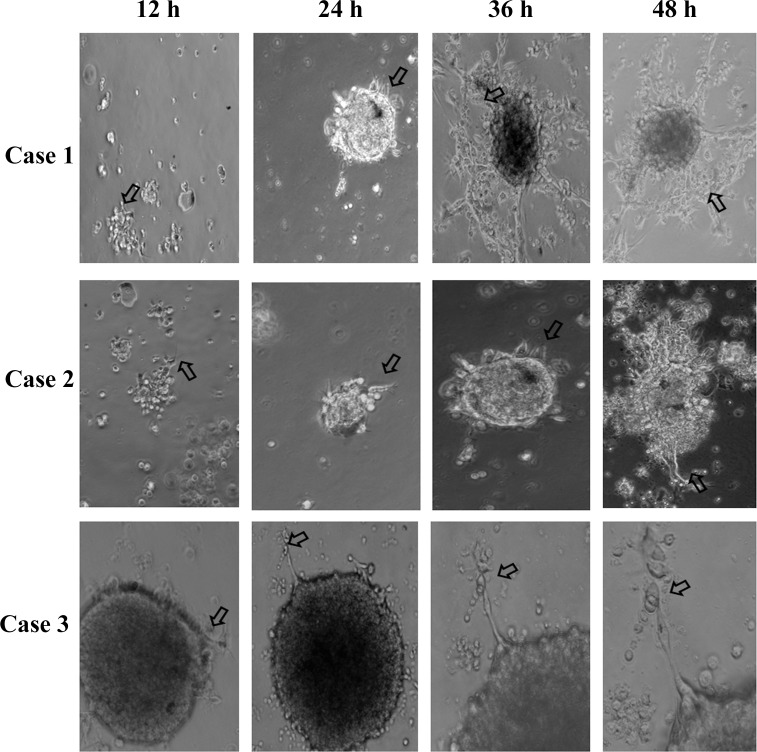
Representative micrographs of the border of the SACC tumor organoids in an ex vivo 3D culture assay was shown. We applied a 3D culture model to observe the action of collective cell invasion with different culture times. The arrow showed the collective invasion cells.

To further detect the role and significance of CK14 in SACC in vitro, we isolated fresh SACC primary tumor tissue and used enzymatic digestion to generate tumor organoids. Tumor organoids had 200–1,000 adherent tumor cells and mirrored the cellular heterogeneity in the SACC primary tumor. Then we cultured tumor organoids in 3D Martigel gels, a model for the microenvironment surrounding invasive cancers [19]. We observed the few of collectively migrating cells emerged from the tumor organoid after 12 h of organoid culture in Martigel gels (Fig 3). Protrusive leader cells could not be found at the front of these invasive strands. After 48 h of organoid culture, the number of the collectively migrating cells augmented. Protrusive leader cells appeared at the front of these invasive strands. At the third and fourth day of culture, there were plenty of the collectively migrating cells. Protrusive leader cells were readily identified at the front of these invasive strands (Fig 3). This confirmed that culture medium containing Martigel can to be applied to observe the action of collective cell invasion of SACC.

**Fig 4:**
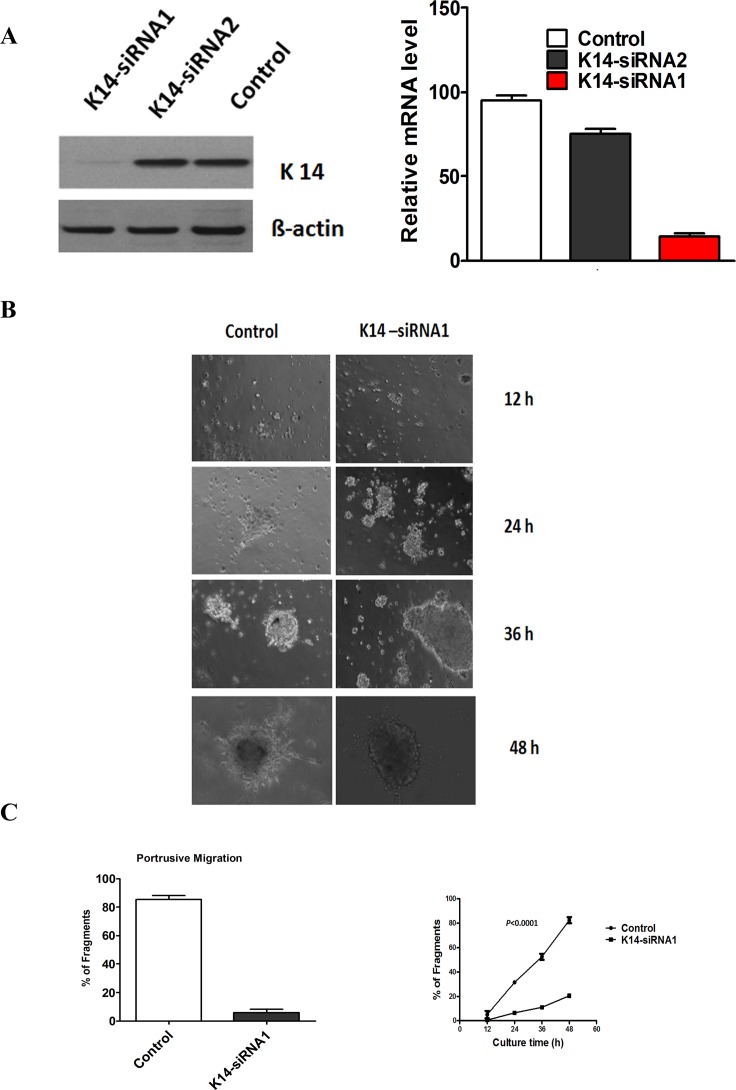
CK14 siRNA receded the collective invasion in an ex vivo 3D culture assay. (A), Left showed Western blotting analysis of CK14 silencing by CK14-siRNA1 and CK14-siRNA in tumor fragments. ß-actin loading control is also shown. Representative of three independent experiments was shown. Right showed transcription levels of CK14 silencing by CK14-siRNA1 and CK14-siRNA2, relative to GAPDH, determined by quantitative RT-PCR. Error bars represent the mean ± SD of triplicate experiments. (B) Representative micrographs of the border of the SACC tumor organoids in an ex vivo 3D culture assay between CK14-siRNA1 and the control was shown. (C) Left showed the number of tumor organoids in control and CK14 siRNA group (*P* = 0.002). Right showed the number of tumor organoids in control and CK14 siRNA group at different time of culture. The data showed that organoids transfected with siRNA-CK14 displayed a significantly lower capability of cells to migrate compared with control at the different times of the culture (*P*<0.0001).

Then, we knocked down CK14 expression by siRNA. We expressed siRNA from 2 different CK14 sequences (siRNA1 or siRNA2) in tumor organoids of SACC. The protein and mRNA levels of CK14 were substantially declined by approximate 80% in CK14-siRNA1 expressing tumor organoids. However, the protein and mRNA levels of CK14 were dropped by about 20% in CK14-siRNA2 tumor organoids (Fig 4A). Thus, CK14-siRNA1 was chosen for further study.

Finally, we observed the collectively migrating cells in tumor organoids transfected with siRNA targeting CK14 and the control. The data showed that there were few of the collectively migrating cells in tumor fragments with siRNA1-CK14, but many of the collectively migrating cells in the control (Fig 4B). Fragments transfected with siRNA1-CK14 displayed a significantly lower capability of cells to migrate compared with the control at the different times of the culture (Fig 4C).The data confirmed that CK14 may contribute to collective cell invasion of SACC.

### CK14 overexpression induced collective migration in 2D monolayer culture

**Fig 5:**
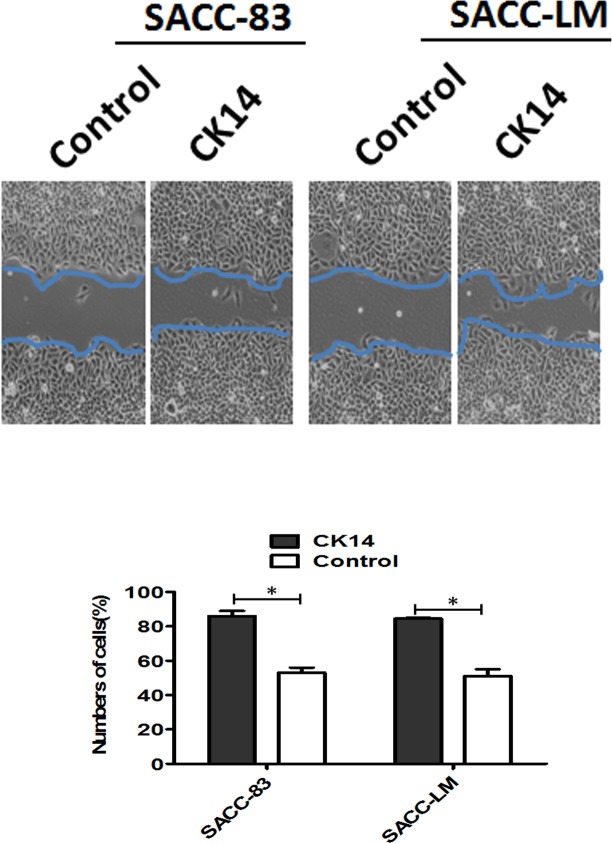
CK14 overexpression induced collective migration in 2D monolayer culture. Serum-starved SACC-83-control and SACC-83-CK14 cell monolayers were cultured and scratched, and they were imaged by phase microscopy after 24 hours. Note that CK14 overexpression significantly induced collective migration in 2D monolayer culture. Error bars represent the mean ± SD of triplicate experiments. The similar results were obtained in SACC-LM cells. *P<0.001.

To further investigate the effect of CK14 on collective migration of SACC in vitro, we stably overexpressed CK14 in SACC-83 cells, as confirmed by immunoblotting (Fig 5A) and real-time PCR. When confluent monolayers were scratched and stimulated with EGF, SACC-83 with CK14 overexpressing at the scratch edge migrated collectively into the wound more rapidly than did SACC-83-control cells within the first 24 h (Fig 5B). In addition, we examined the effect of CK14 overexpressing on the survival and proliferative activity of SACC-83 cells because an increase in survival or proliferation after CK14 overexpressing may also contribute to the migration of SACC-83 cells. The results showed that there was no significant difference of the proliferation between SACC-83 with CK 14 overexpressing and SACC-control in monolayer culture. The similar results were obtained in SACC-LM cells.

## Discussion

Collective cell invasion has been shown to be an important mechanism for normal epithelial formation and cancer invasion [20]. In this study, we analyzed the expression and role of CK14 in the invasion front of SACC patients. The data signified that the positive expression of CK14 in the invasive front was significantly associated with local regional recurrence, distant metastasis and poor prognosis of SACC patients. Then, we observed the phenomenon of collective cell invasion in SACC cells in vitro, and found that in an ex vivo 3D culture assay CK14 siRNA receded the collective invasion and CK14 overexpression induced collective migration in 2D monolayer culture, suggesting that CK14 promoted the collective invasion of SACC.

SACC is located in minor salivary glands (31%), the submandibular gland (14%) and the parotid (2%)[21], and malignant lesions of ACC accounted for a greater percentage of minor salivary gland tumors as compared to major salivary glands [22]. Histopathologically, the tubular and cribriform subtypes of lowergrade malignancy have a better prognosis than the solid subtype of high malignancy [23].In this study, the rate of CK14 positive expression (25/63, 39.68%) in invasive front of minor salivary gland was strongly higher than in major salivary gland (10/58, 17.24%). The CK14 positive expression in invasive front in SACC with tubular or cribriform pattern (19/85, 22.35%) was quite lower than solid pattern (16/36, 44.44%). This indicated that the expression of CK14 seems to involve in the malignancy of SACC. Importantly, the increased levels of CK14 expression were strongly associated with perineural invasion, local regional recurrence, distant metastasis and a poorer prognosis of SACC, and that the patients with positive CK14 had a poorer prognosis than those with negative. These confirmed to the hypothesis that CK14 might be an effective biomarker of collective cell invasion in SACC.

In cancer, collective cancer cell invasion can be detected *ex vivo* in histocultures of tumor explants and 3D matrix-based cell culture of primary melanoma [20, 24]. In the present study of SACC, we found the collective cancer cell invasion in SACC explants and CK14 facilitated the collective cancer cell invasion. This is supported by the reports of Cheung et al [25], who showed that locally disseminated clusters, circulating tumor cell clusters, and lung micrometastases frequently expressed the epithelial cytoskeletal protein CK14. Depletion of CK14 expression abrogated distant metastases and disrupted expression of multiple metastasis effectors. CK14^+^ cells are significantly enriched in breast cancer cells during the phases of metastasis most associated with systemic spread. Conversely, CK14^−^ cells are significantly enriched in the phases of metastasis most associated with proliferation. Intriguingly, CK14^+^ cells were also required for gene expression of multiple metastasis effectors involved in niche remodeling [26–27].

In our previous study, we identified that EMT transcription factors such as Slug, Snail1 and Twist and c-kit promoted the recurrence and metastasis of SACC patients[28–29]. This raised the question whether there was contradiction between collective invasion and EMT in SACC [30]. One possible explanation was that cells can have various degrees of mesenchymal phenotypes, and collective cells might be have epithelial, mesenchymal, or as having an intermediate phenotype [31–33].However, till now, it is unclear what such intermediate phenotypes represent and what advantage they would confer on cells compared with fully epithelial or mesenchymal phenotypes. In SACC, although single invasion and collective invasion are both identified, which one is the predominant phenotype still remains unclear and should be investigated in the future.

Then, what features of tumor progression can regulate the transition from a collective phenotype to a disseminative phenotype? There were some reports focused on this problem to try to elucidate the molecular mechanism of collective cell invasion. Nguyen-Ngoc et al [19] showed that metastatic tumors preferentially disseminated in specific ECM microenvironments, and different effects of different ECM on collective cell migration, indicating the role of ECM in tumor cell dissemination. Chapnick et al [34] demonstrated that TGFβstimulates collective migration primarily through extracellular regulated kinase 1/2 (Erk1/2) activation, showing the upstream determinants and molecular mechanisms behind collective cellular guidance. However, the analysis of the underlying mechanisms of collective invasion in cancer is still at an early stage.

All in all, enforced CK14 expression in SACC increased likelihood of collective cell invasion and associated with a worse prognosis of SACC. More biomarkers of collective invasion should be applied to identify the function and molecular mechanism of collective invasion in more SACC specimens in the future.
